# Lymphoepithelial Carcinoma of the Lung Initially Misdiagnosed as an Intrapulmonary Lymph Node in a Frozen Section: A Case Report

**DOI:** 10.7759/cureus.79263

**Published:** 2025-02-18

**Authors:** Pin-Hao Huang, Yi-Ju Lee, Jeng-Dong Hsu, Yu-Ting Yu

**Affiliations:** 1 Department of Pathology, School of Medicine, Chung Shan Medical University, Taichung, TWN; 2 Department of Pathology, Chung Shan Medical University Hospital, Taichung, TWN

**Keywords:** frozen section, lung pathology, lung tumor, lymphoepithelioma carcinoma of the lung, lymphoepithelioma-like carcinoma, non small cell lung cancer

## Abstract

We report a case of lymphoepithelial carcinoma of the lung (LCL) in a 67-year-old male, which presented as a solitary lesion in the left upper lung, measuring 0.7 cm in diameter. LCL is a rare tumor characterized by a dense lymphocytic infiltrate, often complicating its diagnosis. The patient was initially misdiagnosed as having an intrapulmonary lymph node on the frozen section. However, further immunohistochemical (IHC) staining enabled the pathologist to correctly diagnose LCL. The patient underwent wedge resection and was diagnosed with early-stage I disease (T1N0Mx). There has been no recurrence of the disease one year postoperatively. Clinical, histopathologic, and IHC findings are described to underscore the need for heightened awareness to prevent the misdiagnosis of this condition.

## Introduction

Pulmonary lymphoepithelioma-like carcinoma (LELC) has been redesignated as lymphoepithelial carcinoma of the lung (LCL) and is now recognized as a variant of squamous cell carcinoma according to the most recent 2021 WHO classification [1]. This rare subtype of non-small cell lung carcinoma (NSCLC) is predominantly observed in East Asian non-smokers, with only a limited number of cases reported so far. According to a previous case series in Taiwan, LCL accounts for only 0.9% of all lung cancers [2]. It is strongly associated with Epstein-Barr virus (EBV) infection and exhibits morphologic features identical to those of undifferentiated nasopharyngeal carcinoma (NPC). The characteristic pathological findings include a syncytial growth pattern of tumor cells with large vesicular nuclei, prominent nucleoli, and dense lymphocytic infiltration. Additionally, the tumor size at initial diagnosis varies widely, ranging from 0.7 to 11 cm [3]. We present the first reported case of a small LCL initially misdiagnosed in a frozen section as an intrapulmonary lymph node due to its small size and extensive lymphocytic infiltration.

## Case presentation

**Figure 1:**
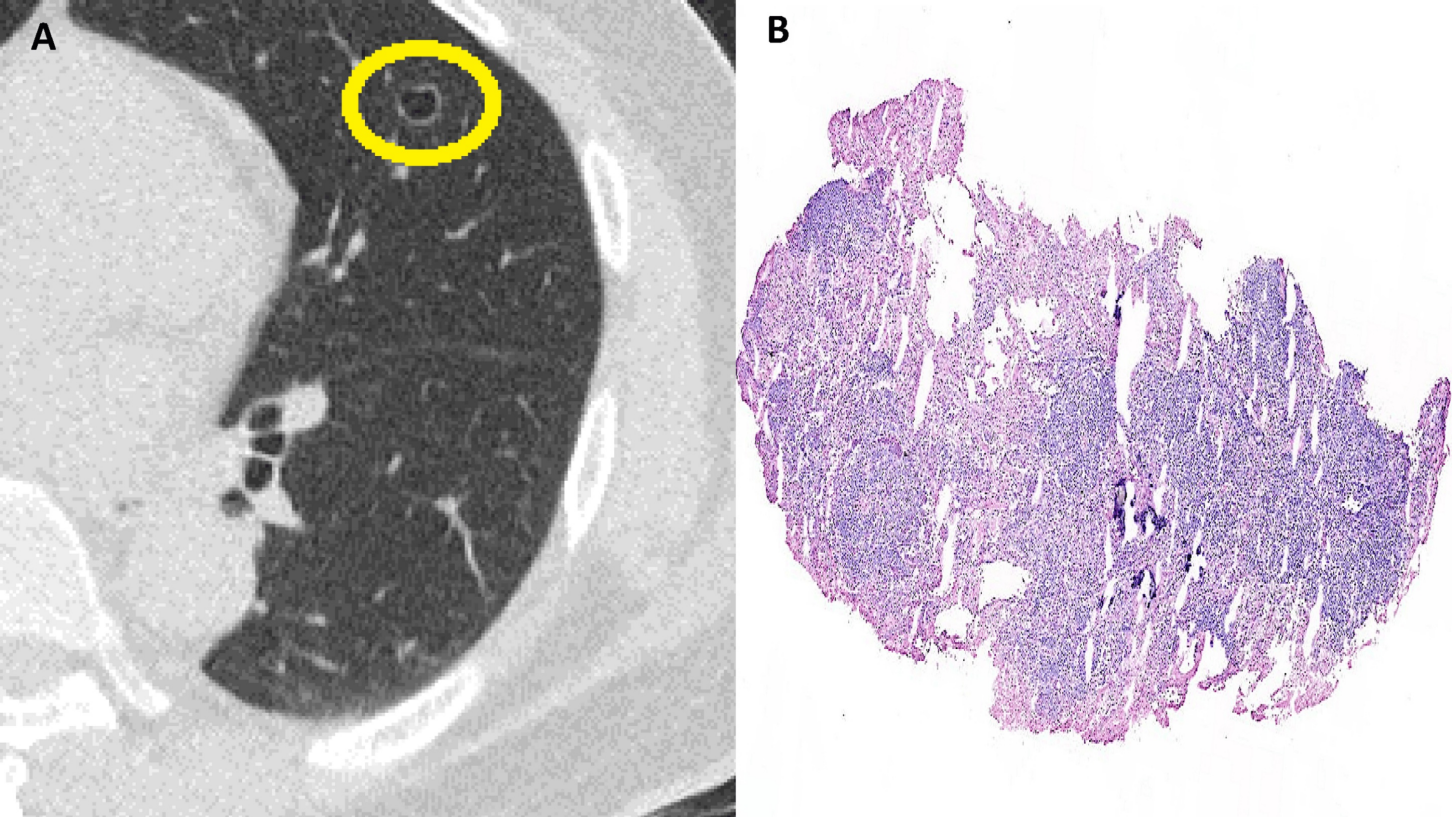
Chest image and intraoperative frozen section (A) CT of the chest showing a cystic lesion at LUL (circle). (B) Intraoperative frozen section revealing a round lesion rich in lymphocytes (original magnification, ×20) CT: computed tomography; LUL: left upper lobe

The patient was a 67-year-old Taiwanese male, a non-smoker with no history of systemic disease. Five years ago, he underwent a self-funded health checkup at a local hospital, which included a chest CT scan. The imaging revealed a 1 cm cystic lesion with associated ground-glass opacities in the left upper lobe (LUL). Radiologists provided differential diagnoses, including benign and malignant entities, infection, trauma, and early-stage lung cancer. The patient remained asymptomatic and was under regular follow-up. Recently, following the death of a family member from lung adenocarcinoma, he decided to proceed with surgical intervention. He was referred to our hospital, where a follow-up CT scan demonstrated a stable 1 cm cystic lesion in the LUL with no significant changes compared to the previous imaging (Figure 1A). Surgery was performed, and an intraoperative frozen section revealed a round lymphocytic lesion, initially diagnosed as an intrapulmonary lymph node (Figure 1B).

**Figure 2:**
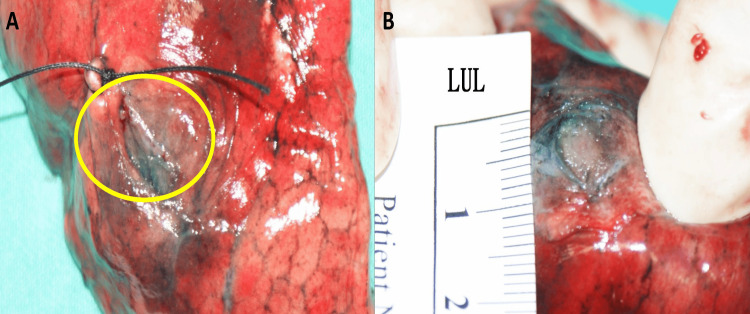
Gross examination of the wedge resection specimen (2A) A cystic, tan-colored lesion (circle). The blue discoloration is due to preoperative CT-guided methylene blue dye localization. (2B) The cut surface of the tumor CT: computed tomography

**Figure 3:**
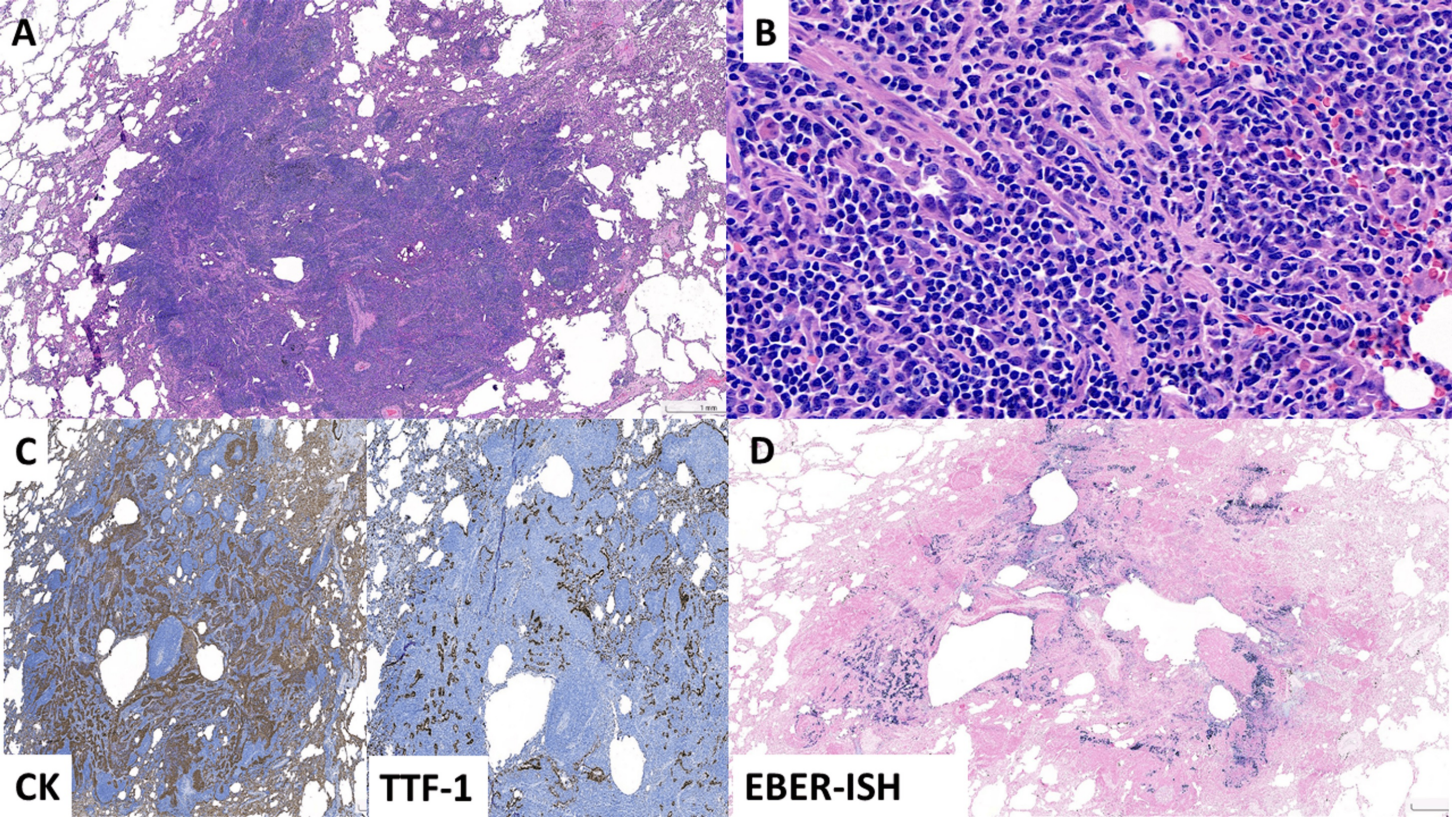
Microscopic findings of the wedge resection (3A) The lesion is round and well-circumscribed. Some lymphoid follicle-like structures are seen (original magnification, x20). (3B) The high-power field shows numerous lymphocytes. The epithelium is not distinctly seen even under high magnification (original magnification, x400). (3C) Anastomosing CK-positive areas and entrapped TTF-1-positive pneumocytes (3D) EBER-ISH demonstrated infiltrating tumor cells in a cord-like pattern CK: cytokeratin; EBER-ISH: Epstein-Barr virus-encoded RNA in situ hybridization; TTF-1: thyroid transcription factor-1

The surgical specimen was obtained via wedge resection. Gross examination revealed a well-circumscribed, tan-colored cystic tumor measuring 0.7 cm (Figures 2A, 2B). The surgical margin was clear, with a distance of 1.3 cm, and the entire lesion was submitted for histopathological examination. Microscopically, the tumor exhibited a dense lymphocytic background (Figure 3A), similar to the frozen section findings. The differential diagnosis by the reviewing pathologist included an intrapulmonary lymph node and mucosa-associated lymphoid tissue (MALT) lymphoma. No abnormal epithelium was distinctly observed under high-power magnification (Figure 3B), and LCL was not initially considered. However, immunohistochemistry (IHC) revealed anastomosing cytokeratin (CK) cocktail AE1/AE3-positive areas (Figure 3C) and entrapped thyroid transcription factor-1 (TTF-1)-positive pneumocytes (Figure 3C). Notably, EBV-encoded RNA in situ hybridization (EBER-ISH) demonstrated infiltrating tumor cells arranged in a cord-like pattern (Figure 3D), even though these tumor cells were not distinctly visible under high-power fields on the hematoxylin and eosin (H&E) stain. The p40 stain (not shown) demonstrated a similar pattern to EBER-ISH. CD3 and CD20 immunostaining revealed a mixed T- and B-cell infiltrate.

The tumor was unlikely to be adenocarcinoma, as it was TTF-1-negative. Although the tumor was positive for the squamous cell carcinoma marker p40, the EBER-ISH positivity supported the diagnosis of LCL. The patient was diagnosed with stage IA1 disease (pT1aN0). As the disease was at an early stage, additional studies, including IHC for programmed cell death-ligand 1 (PD-L1), serum EBV levels, and molecular testing, were not performed. The patient underwent regular follow-up and remained alive and free of cancer one year postoperatively.

## Discussion

LCL is a clinicopathologically distinct cancer previously referred to as pulmonary LELC [1]. According to the updated 2021 WHO classification of thoracic tumors, the diagnostic criteria for LCL include positivity for EBER-ISH, histological resemblance to NPC, and features of poorly differentiated non-keratinized squamous cell carcinoma. LCL accounts for approximately 0.9% of all NSCLC diagnoses [2] and is almost exclusively observed in endemic regions of Asia [1]. Pathologists in East Asia have traditionally equated LELC with lung carcinoma associated with EBV positivity. However, a few cases of LELC in Caucasian patients have been reported in the literature [4-5], with some testing positive for EBER-ISH and others testing negative [6-8]. The latter cases may not align with the updated WHO criteria or the long-standing diagnostic practices in East Asia [6-10].

The term "LELC" has been used to describe tumors in multiple organs that exhibit histological features similar to NPC. However, for tumors arising in other organs, such as the skin, gallbladder, or bladder, the association with EBV has not been well-established in the WHO tumor classification due to the rarity of these diseases. Additionally, the tumor size of LCL at the time of diagnosis varies widely, ranging from 0.7 to 11 cm [3]. Most cases are diagnosed at an early stage and are resectable [4], with survival rates generally better than those observed in patients with other types of NSCLC [5].

Histologically, the tumor is indistinguishable from NPC, typically exhibiting ill-defined cytoplasmic borders with syncytial features. The nuclei are vesicular and contain distinct nucleoli. A characteristic feature is the presence of intense lymphocytic and plasma cell infiltration. Immunohistochemically, the tumor cells express cytokeratins, including the squamous cell lineage markers cytokeratin 5/6, p63, and p40 [3,6]. They are negative for the lung adenocarcinoma marker TTF-1. While the tumor cells do not express T-cell or B-cell markers (CD3 and CD20, respectively), these markers are strongly expressed in the associated lymphoid infiltrate, indicating a reactive process. Most importantly, EBER-ISH is positive [1]. The presented case demonstrated characteristic findings, including an intense lymphocytic and plasma cell infiltrate, positivity for CK, p40, and EBER-ISH, and negativity for TTF-1, consistent with LCL.

The primary differential diagnoses for LCL include NPC, lymphoma, and NUT carcinoma of the lung [1,6]. Accurate differentiation is essential, as lymphoma typically requires nonsurgical management. Misdiagnosis can result in incorrect staging and inappropriate treatment. A thorough examination of the nasopharynx is critical to exclude metastatic NPC [7]. Among pulmonary lymphomas, MALT lymphoma is the most common type of non-Hodgkin lymphoma affecting the lung [3]. Anaplastic large-cell lymphoma can mimic LCL, as it also presents with large tumor cells that have vesicular nuclei, prominent nucleoli, and occasional expression of carcinoma markers [3].

In contrast, NUT carcinoma is characterized by abrupt keratinization and prominent infiltrating neutrophils, with diagnosis confirmed by detecting *NUTM1* rearrangement using molecular methods or NUT-positive immunohistochemistry [1]. In the presented case, the nasopharynx was confirmed to be free of tumor involvement. Cytokeratin positivity supported the diagnosis of carcinoma rather than lymphoma. Additionally, the background of mixed T- and B-lymphocyte infiltrates indicated a reactive process [7]. The characteristic features of NUT carcinoma were absent. Furthermore, EBER-ISH further assisted in confirming the correct diagnosis.

In most cases, intraoperative frozen sections are not submitted. However, some chest surgeons have recently adopted the routine practice of sending small lung lesions for frozen diagnosis [11], as is done in our institute (a topic that will not be further addressed here). In our case, the lymphocytic background impeded an accurate frozen diagnosis. Even under high magnification, the tumor epithelium was not distinctly visible. Hence, the initial differential diagnoses were limited to non-epithelial tumor categories, such as intrapulmonary lymph nodes and MALT lymphoma. CK staining subsequently highlighted the inconspicuous tumor cells, while EBER-ISH provided further assistance in establishing the correct diagnosis. Fortunately, the disease was diagnosed at an early stage, and the misdiagnosis did not affect the treatment plan, as stage I LCL is typically managed with wedge resection [12]. Intrapulmonary lymph nodes are usually identified only after small wedge resections. The postoperative course was uneventful, with no evidence of recurrence observed after one year of follow-up.

Regarding the lymphocytic background, the pathologist’s personal observations suggest that LCL typically contains abundant lymphocytes, with no significant variation in lymphocyte numbers across tumors of different sizes. Moreover, this specific issue has not been addressed in prior research, leaving it unresolved. In this case, the dense lymphocytic background significantly obscured the tumor cells, posing considerable initial diagnostic challenges.

## Conclusions

LCL is a rare disease predominantly observed in East Asia, where EBV infection is endemic. Its pathological and immunohistochemical features, as well as its differential diagnoses, are discussed in this report. In this case, the small tumor size, combined with the dense lymphocytic background that obscured the tumor cells, posed considerable initial diagnostic challenges. LCL should be included in the differential diagnoses of similar cases, particularly in endemic regions. However, variation in lymphocyte numbers across tumors of different sizes has not been explored in prior studies, leaving this specific issue unresolved.
